# Patient-Specific 3D Printed Soft Models for Liver Surgical Planning and Hands-On Training

**DOI:** 10.3390/gels9040339

**Published:** 2023-04-16

**Authors:** Arnau Valls-Esteve, Aitor Tejo-Otero, Pamela Lustig-Gainza, Irene Buj-Corral, Felip Fenollosa-Artés, Josep Rubio-Palau, Ignasi Barber-Martinez de la Torre, Josep Munuera, Constantino Fondevila, Lucas Krauel

**Affiliations:** 1 Innovation Department, Hospital Sant Joan de Déu, Universitat de Barcelona, Santa Rosa 39-57, 08950 Esplugues de Llobregat, Spain; 2 Medicina i Recerca Translacional, Facultat de Medicina i Ciències de la Salut, Universitat de Barcelona, Carrer de Casanova, 143, 08036 Barcelona, Spain; 3 3D Unit (3D4H), Hospital Sant Joan de Déu, Universitat de Barcelona, 08950 Esplugues de Llobregat, Spain; 4 Centre CIM, Universitat Politècnica de Catalunya (CIM UPC), Carrer de Llorens i Artigas, 12, 08028 Barcelona, Spain; 5 Department of Mechanical Engineering, Barcelona School of Industrial Engineering (ETSEIB), Universitat Politècnica de Catalunya, Av. Diagonal, 647, 08028 Barcelona, Spain; 6 Pediatric Surgical Oncology Unit, Pediatric Surgery Department, Hospital Sant Joan de Déu, Universitat de Barcelona, 08950 Esplugues de Llobregat, Spain; 7 Maxillofacial Unit, Department of Pediatric Surgery, Hospital Sant Joan de Déu, Universitat de Barcelona, 08950 Esplugues de Llobregat, Spain; 8 Department of Diagnostic Imaging, Hospital Sant Joan de Déu, Universitat de Barcelona, 08950 Esplugues de Llobregat, Spain; 9 Hepatopancreatobiliary Surgery and Transplantation, General and Digestive Surgery, Metabolic and Digestive Diseases Institute (ICMDM), Hospital Clínic, CIBERehd, IDIBAPS, University of Barcelona, 08950 Esplugues de Llobregat, Spain

**Keywords:** additive manufacturing, material jetting, silicone moulding, liver, surgery, custom-made, personalised medicine, image post-processing, surgical planning

## Abstract

**Background**: Pre-surgical simulation-based training with three-dimensional (3D) models has been intensively developed in complex surgeries in recent years. This is also the case in liver surgery, although with fewer reported examples. The simulation-based training with 3D models represents an alternative to current surgical simulation methods based on animal or ex vivo models or virtual reality (VR), showing reported advantages, which makes the development of realistic 3D-printed models an option. This work presents an innovative, low-cost approach for producing patient-specific 3D anatomical models for hands-on simulation and training. **Methods**: The article reports three paediatric cases presenting complex liver tumours that were transferred to a major paediatric referral centre for treatment: hepatoblastoma, hepatic hamartoma and biliary tract rhabdomyosarcoma. The complete process of the additively manufactured liver tumour simulators is described, and the different steps for the correct development of each case are explained: (1) medical image acquisition; (2) segmentation; (3) 3D printing; (4) quality control/validation; and (5) cost. A digital workflow for liver cancer surgical planning is proposed. **Results**: Three hepatic surgeries were planned, with 3D simulators built using 3D printing and silicone moulding techniques. The 3D physical models showed highly accurate replications of the actual condition. Additionally, they proved to be more cost-effective in comparison with other models. **Conclusions**: It is demonstrated that it is possible to manufacture accurate and cost-effective 3D-printed soft surgical planning simulators for treating liver cancer. The 3D models allowed for proper pre-surgical planning and simulation training in the three cases reported, making it a valuable aid for surgeons.

## 1. Introduction

Pre-surgical simulation-based training with three-dimensional (3D) models has been intensively developed in complex surgeries in recent years, with a significant impact on improving precision medicine [1]. This is also the case in liver surgery, although with fewer reported examples, helping surgeons in tumour resections, tumour volume studies and pre-transplant planning [2,3]. These new simulation-based training techniques can be especially useful in complex paediatric liver surgery cases, helping to navigate the complex hepatic anatomy. However, we found few cases reported in the literature and more evidence is needed for its implementation as an indication process in routine surgical practice.

The simulation-based training involves the use of essential equipment and computer software to model a real scenario. Until now, most approaches in preoperative surgical planning focused on [4,5] live animal and ex vivo models, as well as virtual reality (VR) models. However, these procedures showed different disadvantages. For instance, animals and ex vivo models do not really mimic the human body, and there are increasing ethical concerns regarding their use [5,6]. VR models, despite having advanced significantly in recent years and representing a cheaper alternative to 3D printed models [6], do not offer physical and tactile interaction and, hence, the training is unrealistic.

Surgical planning models, also known as phantoms, are patient-specific anatomical replicas that surgeons use before performing the operation, guiding them through the complex anatomy to define the surgical approach and enhance their performance. In the case of the liver, these surgical planning prototypes are manufactured for two reasons: (1) visualisation, in which these prototypes are manufactured in order to study the different anatomical structures, a key factor for achieving a successful operation; and (2) hands-on training, which is also known as simulation-based training, oriented towards the preoperative planning, selection of the optimal surgical approach and reduction of potential surgical complications [2]. This normally requires soft materials to better mimic the biomechanical properties of tissue, especially for the organ in question. However, determining and quantifying the mechanical behaviour of soft biological tissues is challenging due to their intrinsic labile nature [7,8,9]. The mechanical properties of the tissues can change due to various factors, such as age, amount of fat, and death, and there is still no consensus on the mechanical behaviour values of certain tissues, such as liver cells [7,8,10,11].

Manual dexterity is one of the skills improved by using surgical planning prototypes, more specifically, the application of the correct amount of force in the surgical procedure since surgical simulation revealed that more than 50% of errors are attributable to excessive force [12]. In general, novice surgeons apply more force than necessary in comparison with experienced surgeons. Considering the data [13], the average force applied is mainly around 0.5 N, although at specific moments, such as gripping tumour tissue, it might reach 1.25 N. 

The use of 3D physical models can change this paradigm and make a difference. For their manufacture, 3D printing and the AM process can be used. This can be defined as the procedure of joining materials to make objects from a digital 3D model data, normally layer-upon-layer (each one has a thickness of 0.001 to 0.1 inch [14] through a series of cross-sectional slices, as opposed to subtractive manufacturing technologies [15]. 

The AM technologies can be classified into seven categories according to the ISO/ASTM 52900 Standard [15]: binder jetting, direct energy deposition (DED), material extrusion (including fused filament fabrication (FFF) and paste/slurry-based extrusion, also known as direct ink writing (DIW)), material jetting, powder bed fusion (including selective laser sintering (SLS) and selective laser melting (SLM)), sheet lamination and vat photopolymerisation (including stereolithography (SLA), digital light processing (DLP), and volumetric additive manufacturing (VAM)).

In recent years, several studies have manufactured different realistic 3D models: kidney [4], brain [16], cardiac [17,18], neuroblastoma [19], liver [2,3,4,20,21,22,23,24,25], etc. However, most of the liver cases found in the literature had limitations, such as the accuracy of the 3D model and lack of quantitative quality validation in the production process. (1) Souzaki et al. [22] and Zein et al. [23] manufactured liver cases using the material jetting technology, which is very expensive and limits its use in hospitals. (2) Witowski et al. [24] reported a more cost-effective approach but presented challenges in the anatomical accuracy of the final model. (3) Tan et al. [4] developed a liver case taken from an online database for surgical simulation training and, therefore, the case was probably not used for preoperative surgical planning. Finally, (4) in most of the published cases, there is no quantitative validation of the models [21,22,23]. However, Tejo-Otero et al. [21] were not only able to obtain a 3D physical model but also represent the mechanics of the liver through the use of different hydrogels and silicones.

Amongst the different materials that have been used for the present application, hydrogels and silicones appear as the most common soft materials. On the one hand, hydrogels are soft materials that are mainly used in tissue engineering applications, although they have been used in some prototypes [16,21]. Nevertheless, they have some disadvantages: (1) a lot of preparation and processes are necessary; for example, Forte et al. had to perform one or two freeze-thaw cycles; (2) they are not consistent enough, posing challenges in the manufacturing and repeatability, as reported by Tejo-Otero et al. [21]; and (3) they might degrade very quickly compared to silicone-made prototypes. One of these examples is cellulose, commonly used in certain bio-applications, yet not the best for the present study for the reasons mentioned above [26,27]. On the other hand, silicones are a synthetic polymer made up of silicon, oxygen, carbon and hydrogen [28]. They are very versatile materials that can be formulated into various types of materials, such as elastomers, gels (when it is in a semi-solid state) or adhesives, showing the possibility of being used in a wide range of applications [28]. They are of interest in the biomedical field due to different factors [28] depending on the target use: (1) they are firm and flexible; (2) stable to temperature and chemical conditions; and (3) they are inert and non-toxic. In this way, they are widely used in the biomedical field in a wide range of applications, such as breast implants [29], prostheses [30], hypertrophic burn scars [31] or phantoms [32]. The latter application can be performed with hydrogels, although, as mentioned before, they are not very consistent, and their lifespan is very short, not enough to be used for purposes apart from surgical planning, such as patient education [33] or for medical school interns. 

**Figure 1:**
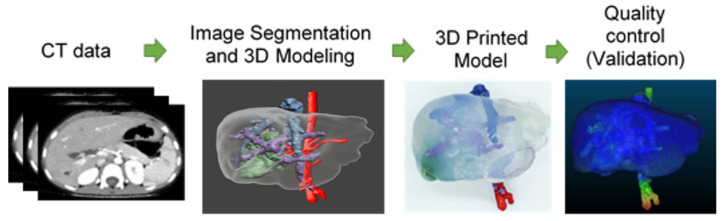
Graphical abstract representation of the high-level process followed in cases presented in this research.

Therefore, the aim of the present article is to report the full process of three additive-manufactured liver models as an aid in the surgical planning of three complex paediatric liver cases by explaining the complete workflow for the development of each case, namely: (1) medical imaging acquisition; (2) segmentation; (3) 3D printing; (4) quality control/validation; and (5) cost. Figure 1 shows the process of the present research study. 

## 2. Results and Discussion

### 2.1. Results

**Figure 2:**
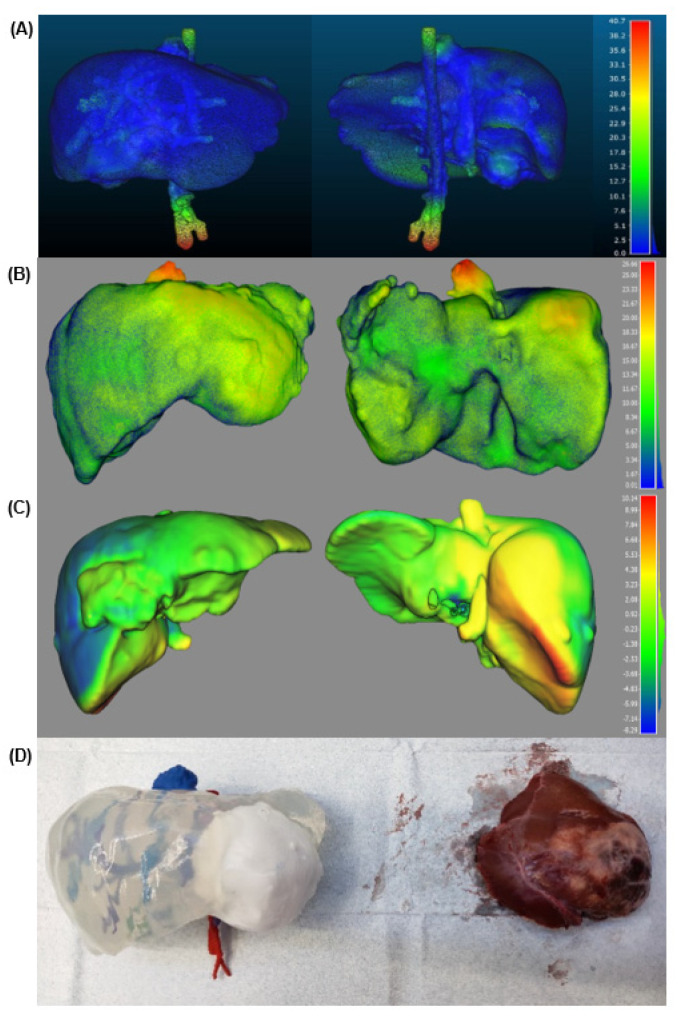
Quantitative analysis of the surgical planning prototype. The real organ 3D model scan was used as the reference point. The surface colour of the phantom model represents the distance error. (**A**) Case #1. (**B**) Case #2. (**C**) Case #3. The distance is measured in mm. (**D**) Comparison between the prototype of case #2 and the tumour after its removal.

The 3D physical models were manufactured before the planned surgery, so the surgical team was able to prepare the case, simulate and practice using the models in advance. The prototypes were a 1:1 scale of the patient’s organs and, consequently, gave an impression of what to expect during surgery. This can be confirmed by the comparison of the tumour removed from case #2 (see Figure 2D). Additionally, the soft consistency of the prototype permitted the use of the surgical instruments that would be used in performing the operation. In summary, the models gave surgeons a new tool for surgical planning and pre-surgical simulation training.

#### 2.1.1. 3D Models

**Figure 3:**
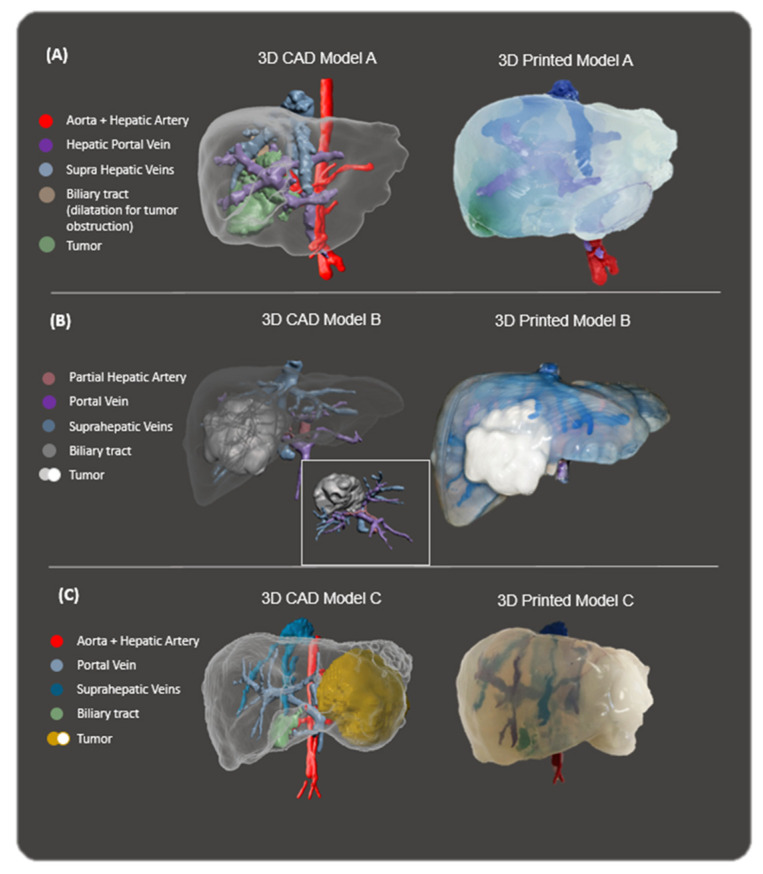
3D models in CAD file and manufactured final model of the different cases: (**A**) Case #1. (**B**) Case #2. (**C**) Case #3.

Figure 3 shows the surgical planning prototypes of the hepatobiliary oncological cases. The silicone used gives the desired transparency that surgeons are looking for, which is very difficult to achieve with other types of hydrogels or silicones. The transparency gives surgeons the advantage of observing the exact position of internal blood vessels and their relation to the tumour and anatomy. Moreover, as soft silicone was used, it was possible to practice with it by using medical surgical tools such as lancets or Kelly forceps with surgical sutures. 

#### 2.1.2. Validation

There is no significant difference between the original organs and the phantoms (see Figure 2). Regarding Cases #1 and #2, the biggest error was measured in the blood vessels located outside the phantoms. This happened because it was not possible to scan those parts. Therefore, these areas appear in red, which indicates a higher error. Table 1 summarises the parameters obtained from the validation of the three cases. All in all, the dimensional error is low, less than 3.35 mm, 4.74 mm and 2.1 mm for each case, respectively. Apart from a CT validation, the usefulness and accuracy of the 3DP replicas can be clinically validated by comparing them at the time of surgery with the removed specimen (see Figure 2D). These prototypes overcame the drawbacks of the previous ones for several reasons: (1) the use of transparent silicone, using highly accurate casting techniques thanks to the defined production process and the 3D technologies providing good visual feedback of the hepatobiliary anatomy; (2) this prototype was used for last minute enquiries during the operation thanks to the use of sterilisable materials; and (3) the surgeons appreciated the liver softness in surgical planning, compared to other previously used prototypes.

#### 2.1.3. Cost

Table 2 summarises the cost in terms of materials and labour. The labour cost is the combination of engineers for the segmentation and 3D printing, as well as the necessary post-processing. Regarding oncologists and radiologists, time is not taken into consideration since their work is something commonly performed in operations. It can be highlighted that this price is lower than that of prototypes manufactured using other techniques, such as material jetting, which may cost 2000 euros (€). 

#### 2.1.4. Time

Table 3 shows the different times needed for each part of the process. The 3D printing parts in the present table show the time of the 3D model, although it must be taken into consideration that most of these parts are 3D printed with other works needed for other applications or clients. In the SLS part, post-processing is also considered.

### 2.2. Discussion

This study demonstrates that it is possible to manufacture 3D-printed soft surgical planning prototypes for a better simulation experience. For the present research, three different cases were taken into consideration, in which the full production process was included: from the DICOM acquisition to their manufacture, as well as a final validation and summary of costs. 

The silicones used, as mentioned, are softer than the common materials used in 3D printing techniques, such as PLA or PA12. One of the silicones used had a value of 38 Shore A, which is a soft value, although it is not as soft as the liver tissue. According to different studies, the measured liver tissue is in the range of Shore 00 [7,10,11]. Although in this sense, it is possible to achieve 100% mechanic mimicking, which is the best option when hydrogels are not used. Additionally, silicones, like the ones used, offer the possibility of seeing the inner anatomical structures, which are not possible to be seen during the operation as well as with other types of materials. 

Aside from this enhanced soft texture simulation experience, it is possible to use these models to improve patients’ experience before an operation, thanks to the better understanding they manifest when viewing and touching the 3DP replicas, as opposed to only biplanar CT images. Additionally, as an indirect 3D printing technique was used, it is possible to reuse these moulds for manufacturing more prototypes in the future for further teaching purposes.

The results of the validation of the three cases are in concordance with Bücking et al. [34], which measured an error of 1.3% between the phantom model and the patient’s liver. Moreover, Witowski et al. [35] measured an average error of around 2 mm, which is similar to the results achieved in the present prototypes. Regarding the validation of the prototypes, other medical imaging techniques could be used aside from CT scanning [36]: positron emission tomography (PET), single-photon emission computed tomography (SPECT), magnetic resonance imaging (MRI), ultrasound (US), and mammography. Amongst the previously mentioned medical imaging techniques, the least used for phantom imaging is mammography [36]. CT is shown to be the best due to its good spatial resolution, high contrast and signal-to-noise ratio, which enhance the differentiation of the anatomical structures [37]. Additionally, CT with MRI are the most used for quantitative results measurements; however, Mitsouras et al. [38] concluded that MRI demonstrated larger differences in the phantom compared to the CT data. 

The surgical planning prototypes, which were manufactured by printing a mould in PLA using FFF or PA12 using SLS, silicone casting and manufacturing PA12 with SLS for the internal parts, had a total cost of approximately €500. For Madurska et al. [39], manufactured using TangoPlus^®^ and TangoBlack^®^ (Stratasys, Rehovot, Israel) without casting or moulding, the cost ranged between USD$500–600; it was approximately USD$1000 in Igami et al. [40] or more than USD$2000 per model in Prashanth et al. [41]. This was more expensive than our prototypes if only the material costs were taken into account. On the other hand, Witowski et al. [24] manufactured using silicone and FFF. In this case, the total cost was around USD$130, which represents a similar cost to our prototype in terms of material costs. The production cost of the mould with SLS technology and PLA material of case #2, as an alternative to the PLA moulds produced by FFF in the other two cases, has been more expensive without providing a significant advantage in the manufacturing process or in the quality of the final result.

#### 2.2.1. Clinical Relevance

The definition and implementation of a digital workflow for pre-surgical simulation training and planning using 3D printing technologies in the treatment of patients with liver cancer represent a promising and unique opportunity. These new techniques allow for a better understanding of complex anatomy and a first approach to 3DP replicas that allow surgical dissection and surgical rehearsal. This opens the way for surgeons in training to better prepare for very complex surgeries. Moreover, although the integration of this new multidisciplinary approach requires an increased turnaround time in planning, and there is far less experience with soft tissue 3DP than with bone structures, there is some data demonstrating a reduction in surgical time, complications and outcomes [2].

#### 2.2.2. Limitations

The major technical drawback of the manufactured 3D models is that both the blood vessels and the tumour have a rigid consistency. Nevertheless, they were an excellent option for surgeons for preoperative surgical planning and simulation training. This is a new technological development, and research is ongoing in our laboratories to improve the prototypes by using more elastic parts for all anatomical structures. The next step is to combine the soft-tissue-mimicking results with current prototypes [7].

## 3. Conclusions

We demonstrate that it is possible to manufacture accurate 3D-printed soft surgical planning prototypes at a low cost compared to the existing alternatives. The 3D models allowed for proper pre-surgical planning and simulation training in the three cases reported, being a valued aid for surgeons. Furthermore, we present a detailed workflow for extending the hospital production of 3D pre-surgical liver models. This full process could also be used in other medical indications or areas, such as neuroblastomas, traumatology or brain tumours. In the future, new materials with advanced properties, such as hydrogels or the combination of different materials (silicones and hydrogels), could also be used for the different anatomical parts, improving tissue mimicking. Another possible approach is the manufacture of multi-material 3D prototypes using a hybrid multi-material 3D printer, in which filaments and slurry-based materials, such as hydrogels and silicones, could be combined.

## 4. Materials and Methods

### 4.1. Cases Presentation

Three paediatric cases were transferred to a major paediatric referral centre for treatment evaluation. All three patients presented complex hepatic tumours. Case #1 was a 2-year-old male. After radiological evaluation using contrast CT (computed tomography) scan and MRI (magnetic resonance imaging) (see the section below), a hepatic mass with biliary tract dilatation was observed. Drainage and a tru-cut biopsy were performed, with a diagnosis of biliary tract rhabdomyosarcoma. Chemotherapy was given according to the recommended SIOP protocol, and after a good response, surgery was advised. Case #2 was a 1-year-old female with a left hepatic mass and alpha-feto protein elevation. Biopsy confirmed Pretex II hepatoblastoma, and surgery was advised. Case #3 was a 1-year-old male presenting a hepatic mass compatible with hepatic hamartoma. (See Table 4). 3D reconstructions and 3D printed models were performed for surgical planning purposes. Table 4 summarises the patients’ information. 

### 4.2. Digital Workflow for 3D Printing Pre-Surgical Simulation-Based Training

**Figure 4:**
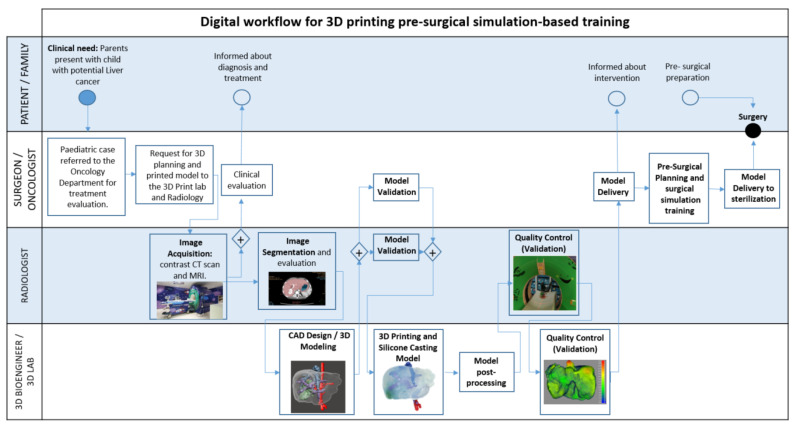
Process flowchart representation of the digital workflow for 3D printing pre-surgical simulation-based training.

The pre-surgical 3D planning and simulation based-training requires a multidisciplinary team and a cross-functional alignment of surgeon, oncologist, radiologist and 3D planning bioengineer. The summarised process workflow is defined and presented in the flowchart below (See Figure 4):

The following sections describe the main stages of the process in more detail.

### 4.3. Image Acquisition

The first step is to obtain information about the anatomy, geometry and tissue composition of the corresponding normal and pathological structures. Radiological multiplanar imaging, such as CT or MRI, provides the most useful information, not just about the findings (e.g., localisation, number of lesions, etc.) but also allows the segmentation of different structures.

**Figure 5:**
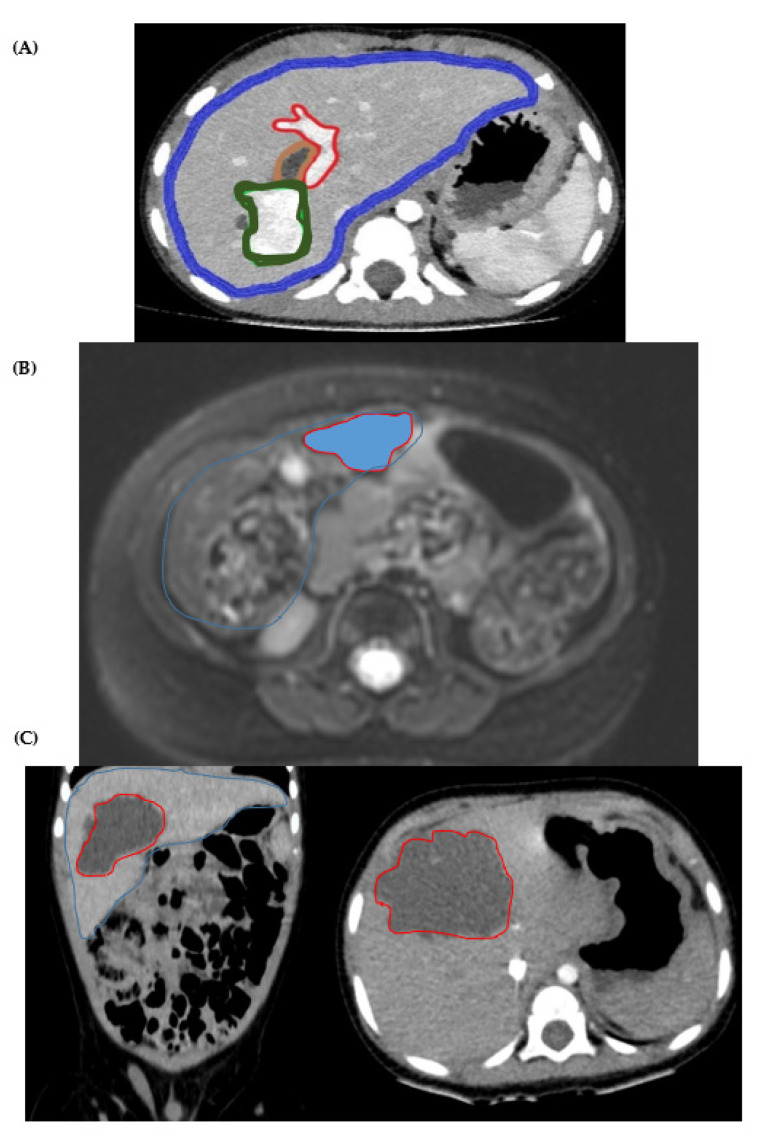
(**A**) CT image with the different anatomical parts highlighted: (1) Liver is outlined in blue; (2) Portal system is circled in red; (3) Dilated intrahepatic biliary tract (tumour) is outlined in brown; and (4) the rest of the tumour, which originates in the biliary tract, is circled in green. (Left) Coronal or frontal plane. (Right) Axial plane. (**B**) MRI of the case (Axial plane). The tumour is contoured in red. The liver is contoured in blue. (**C**) Left: MRI of the case (Frontal plane). Right: MRI of the case (Axial plane). The tumour is contoured in red. The liver is contoured in blue.

The patient’s livers were scanned using computed tomography (iCT 256 Philips) with a standard paediatric abdominal CT protocol. In all cases, the abdominal CT was performed following these parameters: 1mm slice thickness with 0.5 reconstruction, 80 kV, exposure modulation and IMR reconstruction (See Figure 5). The CT protocol included iodinated contrast injection (split of standard weight/dose) to obtain in one acquisition a double vascular image (arterial and venous-portal). To complement the CT acquisition, an MRI study was added to evaluate the tissue features. The protocol included T2, DWI and axial post-contrast dynamic 3D T1. 

### 4.4. Image Segmentation and Surface Reconstruction

The images acquired using the CT and MRI techniques were saved in DICOM (digital imaging and communications in medicine) format (Figure 5). The image segmentation was carried out by an expert radiologist to extract the anatomy to be used in the 3D planning and printing. A semi-automatic segmentation was performed using the IntelliSpace Portal© software from Philips© (Amsterdam, The Netherlands). The segmentation process helps obtain the 3D surface STL model with the addition of the DICOM segmented mask regions of interest and is exported to an STL file.

**Figure 6:**
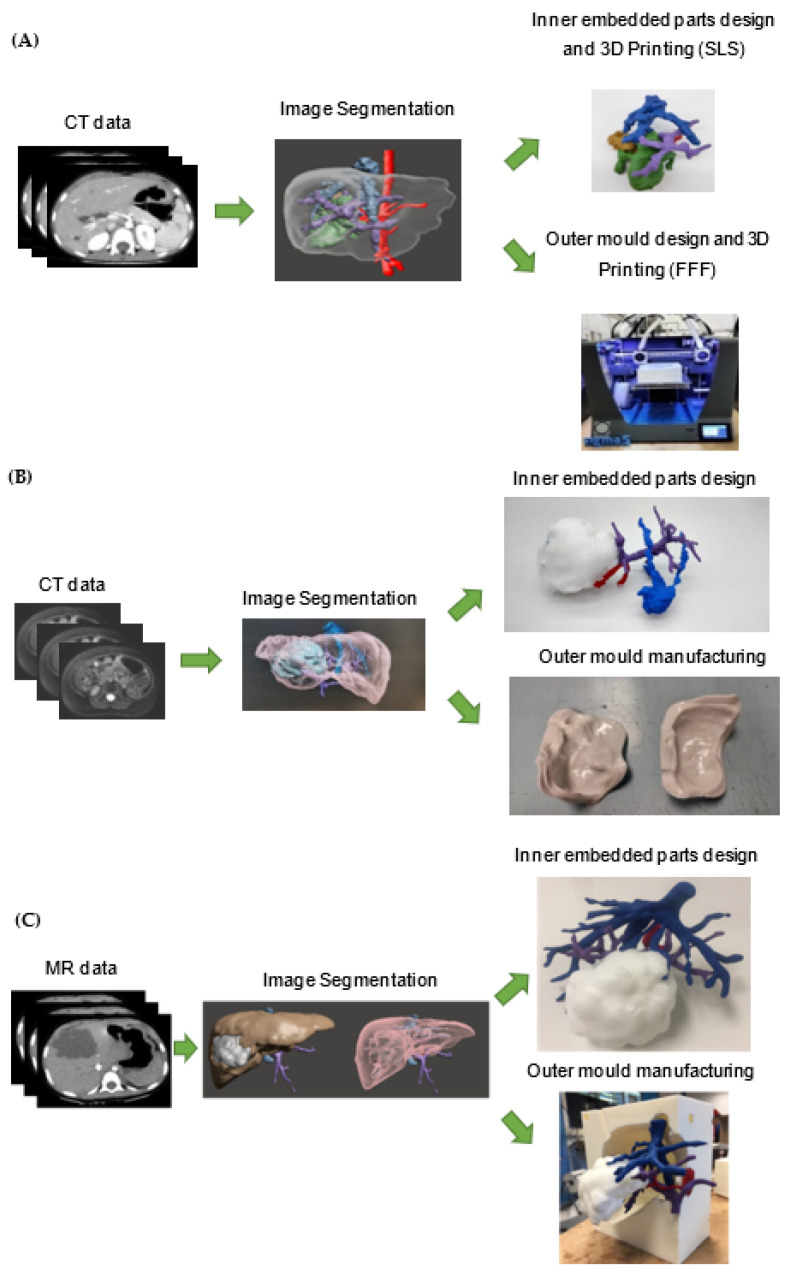
Workflow for the design of the 3D model. (**A**) Case #1. (**B**) Case #2. (**C**) Case #3.

The workflow of the surgical planning prototypes can be seen in Figure 6. The different parts of the prototypes are highlighted in different colours so that the different anatomical structures are clearly distinguished. Regarding case #1, the part of the image segmentation of Figure 6A depicts the different anatomical parts: (1) the aorta and hepatic artery are in red, (2) the portal vein is in purple, (3) the vena cava and supra-hepatic veins are in blue, (4) the biliary tract can be found in brown, dilated by tumour obstruction, and finally, (5) green corresponds to the tumour. Case #2 in Figure 6B shows: (1) the portal vein in purple, (2) the vena cava and supra-hepatic veins in blue and (3) the tumour in light blue. Case #3 in Figure 6C shows: (1) the portal vein is in purple, (2) the vena cava and supra hepatic veins are in blue and (3) the tumour is grey.

### 4.5. CAD Design and 3D Moulding

The extracted 3D STL files were transferred to the 3D bioengineering specialists in order to create computer-aided design (CAD) files. The 3D models of each case were created using Autodesk Meshmixer©/MeshLab© software (San Rafael, CA, USA). After clinical validation of the 3D model reconstruction by a senior paediatric oncology surgeon and a senior radiologist, the virtual simulation of the procedure and calculation of the potential tumour volumes were made. Next, the preparation of the different anatomical parts to be printed was begun. In these cases, in making the models, it was decided to opt for combining 3D printing and material casting for the liver tissue with rubber-based materials to reduce the cost of the model and better mimic liver parenchyma characteristics.

### 4.6. 3D Printing and Silicone Casting of the Phantom

**Figure 7:**
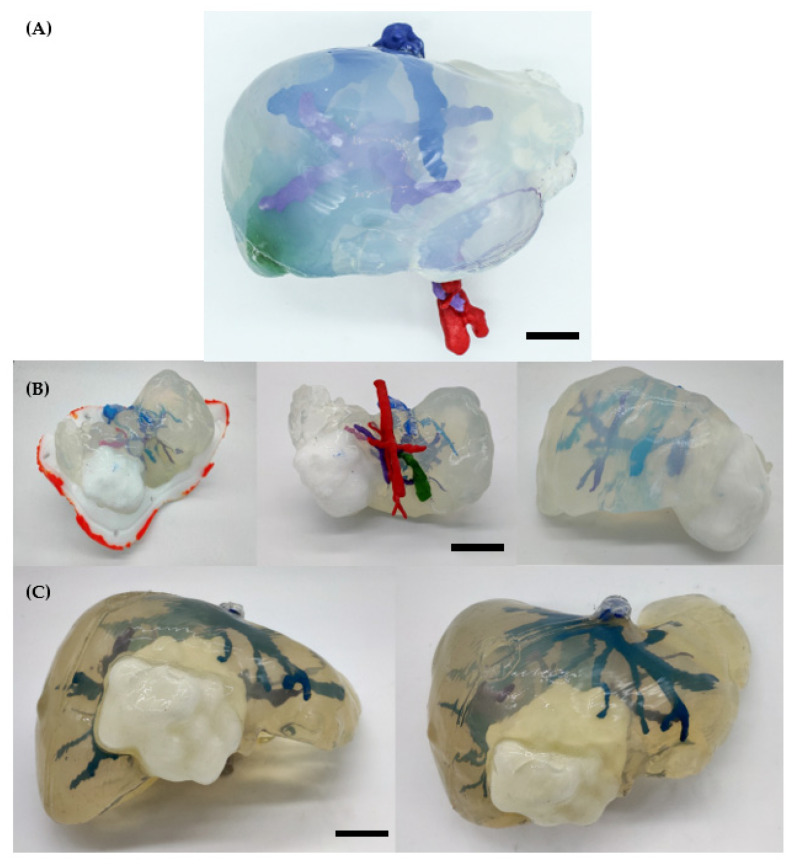
Surgical planning prototypes. (**A**) Case #1: Rhabdomyosarcoma (bar corresponds to 3 cm). (**B**) Case #2: Hepatoblastoma (bar corresponds to 5 cm). (**C**) Case #3: Mesenchymal Hamartoma (bar corresponds to 3 cm).

For the 3D printing of the surgical planning prototype, the moulding technique was used, in which a material is cast inside a 3D-printed mould. The embedded inner anatomical parts (vessels, tumours, biliary tract, etc.) were manufactured using 3D printing SLS technology and PA 12 material (Figure 6). The 3D printer used was a Ricoh AM S5500P at CIM UPC facilities, which has a layer thickness of 0.08–0.1 mm, displayed in high resolution. PA 12 was used since it is the best option in order to place rigid parts, like the inner embedded structures (veins, tumour, biliary tract), precisely inside the mould when the material is cast. Once these inner parts are 3D printed, they are coloured so that the different anatomical structures can be distinguished. Regarding the mould, two different approaches were carried out. For cases #1 and #3, the outer mould was manufactured using a polylactic acid (PLA) filament in FFF. The 3D printer used was a Sigma model (BCN3D Technologies, Barcelona, Spain), which offers a dimensional precision of ± 0.2 mm and can achieve a layer thickness of 25 µm. For case #2, the outer mould was manufactured using PA12 with the SLS technology. The materials, as well as the manufacturing process, were changed in order to show the possibility of using different technologies and assess their impact both economically and in terms of quality of the final model. Once all parts are 3D printed, they are assembled, and the silicone is cast. The commercial silicone used is Essil 291 Resin–38 Shore A at a volume ratio of 10:1 with a catalyst (ESSIL 292 Catalyser) for all cases (see Figure 7). According to Curtis et al. [42], silicone gels are normally supplied in a two-part fluid system and cured through a platinum-catalysed addition reaction. Parts A (in this case Essil 291 Resin) and B (in this case ESSIL 292 Catalyser) are mixed at a desired ratio (in this case 10:1) and cured (usually by exposure to elevated temperature) to yield a sticky but cohesive mass. 

### 4.7. Validation

CloudCompare© V2.11 was used for the validation of the surgical planning prototypes. The printed anatomical models were compared against the computer-aided designed models to assess printing accuracy [20]. The printed models were scanned and segmented using the same acquisition and segmentation technique as the original cases, obtaining the STL files. Both STL files of each case (the one obtained from the initial patient acquisition and the one from the 3D-printed model) were aligned by selecting different referential points in each mesh. Then, the distance between both meshes was computed using the cloud-to-cloud distance (Hausdorff distance algorithm), which is a dimensional measurement for comparing image segmentations between two set points [43].

## Figures and Tables

**Table 1:** Results of the validation.

Case	Parameters	Values [mm]
#1	Average Distance	3.35
	Standard deviation	2.2
	Maximum Distance	40.7
#2	Average Distance	4.74
	Standard deviation	4.16
	Maximum Distance	26.37
#3	Average Distance	2.1
	Standard deviation	1.98
	Maximum Distance	10.03

**Table 2:** Cost of the surgical planning prototypes.

Case	Process	Material	Material Cost [€]	Labour Cost [€]	Total [€]
#1	SLS	PA 12 SLS (Inner Parts)	112	138	250
	FFF	PLA FFF (Outer Mould)	16	133	149
	Moulding	Essil 291 Resin	12	113	125
	**Total [€]**		**140**	**384**	**524**
#2	SLS	PA 12 SLS (Outer Mould)	206	140	458
		PA 12 SLS (Inner Parts)	112		
	Moulding	Essil 291 Resin	12	113	125
	**Total [€]**		**330**	**253**	**583**
#3	SLS	PA 12 SLS (Inner Parts)	130	138	268
	FFF	PLA FFF (Outer Mould)	14	133	147
	Moulding	Essil 291 Resin	12	113	125
	**Total [€]**		**156**	**384**	**540**

**Table 3:** Time taken in each step of the process following the digital workflow for 3D printing pre-surgical simulation-based training of the surgical planning liver models.

Case	Segmentation	CAD Modeling	3D Printing		Silicone Casting	Total [Hours]
#1	45 min	20 min	SLS (Inner Parts)	240	10 min	16/24 (One full day)
			FFF (Outer Mould)	720 (Overnight)		
#2	38 min	25 min	SLS (Inner Parts)	240	10 min	8 (One working day)
			SLS (Outer Mould)	240		
#3	42 min	20 min	SLS (Inner Parts)	240	10 min	16/24 (One full day)
			FFF (Outer Mould)	720 (Overnight)		

**Table 4:** Patients’ Information.

	Age (Years)	Sex	Diagnosis	Surgical Approach
Case #1	2	M	Biliary tract Rhabdomyosarcoma	Extended Right Hepatectomy andRoux-en-Y Hepaticojejunostomy
Case #2	1	F	PreText II Hepatoblastoma	Left Hepatectomy
Case #3	1	M	Mesenchymal Hamartoma	Tumorectomy (segment IV)

## Data Availability

Due to the sensitive nature of the data used in this study, the datasets used and/or analysed during the current study are available from the corresponding author on reasonable request. Imaging data will remain confidential and will not be shared.
